# 18. TRACKING THE MECHANISMS UNDERLYING WORKING MEMORY DYSFUNCTION IN SCHIZOPHRENIA FROM CORTICAL MICROCIRCUITS TO THE SYSTEMS LEVEL

**DOI:** 10.1093/schbul/sby014.069

**Published:** 2018-04-01

**Authors:** Robert Bittner

**Affiliations:** University Hospital Frankfurt, Goethe University

## Abstract

**Overall Abstract:** Converging behavioural and neuroimaging evidence indicates that an inability to regulate behaviour by working memory (WM) is a core feature of people with schizophrenia (PSZ) which significantly influences their level of recovery. WM dysfunction has also gained interest as a target of cognitive enhancement interventions and as an intermediate phenotype in the study of the genetic architecture of schizophrenia. These translational strategies critically depend upon a clear understanding of the underlying cognitive and neurophysiological disturbances. The aim of the symposium is to highlight current approaches to identify the pathophysiological mechanisms underlying WM deficits on both the microcircuit and the systems level.

M. Ichinose will report cognitive modelling data showing that PSZ have elevated internal noise during perceptual processing, which was inversely correlated with WM precision. This indicates that ‘noisy’ perception contribute to impairments in WM and other cognitive domains.

R. Bittner will present behavioural data from PSZ and imaging genetics fMRI data from a large cohort of healthy participants. The results of these studies indicate, that specific impairments in bottom-up attentional processes associated with genetic risk contribute to the dysfunction of working memory encoding in schizophrenia.

A. Anticevic will present computational microcircuit models, resting state fMRI data and fMRI data using ketamine which provide evidence for a disturbed excitation-inhibition (E-I) balance and resulting in large scale dysconnectivity in schizophrenia in the context of working memory and other cognitive processes.

The research presented in this symposium integrates computational, neuroanatomical, electrophysiological, pharmacological, genetic, behavioural and neuroimaging methods to connect pathophysiological mechanisms at the microcircuit level such as increased neuronal noise and an abnormal E-I balance to disturbances in large scale brain networks at the systems level and to behavioral impairment. Such an integrative approach promises to yield new insights into the pathophysiology of cognitive dysfunction in schizophrenia.

